# Dietary rescue of lipotoxicity-induced mitochondrial damage in Peroxin19 mutants

**DOI:** 10.1371/journal.pbio.2004893

**Published:** 2018-06-19

**Authors:** Julia Sellin, Christian Wingen, Dominic Gosejacob, Deniz Senyilmaz, Lea Hänschke, Sven Büttner, Katharina Meyer, Daniele Bano, Pierluigi Nicotera, Aurelio A. Teleman, Margret H. Bülow

**Affiliations:** 1 University of Bonn, Life & Medical Sciences Institute (LIMES), Molecular Developmental Biology, Bonn, Germany; 2 German Cancer Research Center, Signal Transduction in Cancer and Metabolism, Heidelberg, Germany; 3 German Center for Neurodegenerative Diseases (DZNE), Bonn, Germany; Baylor College of Medicine, United States of America

## Abstract

Mutations in peroxin (PEX) genes lead to loss of peroxisomes, resulting in the formation of peroxisomal biogenesis disorders (PBDs) and early lethality. Studying PBDs and their animal models has greatly contributed to our current knowledge about peroxisomal functions. Very-long-chain fatty acid (VLCFA) accumulation has long been suggested as a major disease-mediating factor, although the exact pathological consequences are unclear. Here, we show that a *Drosophila Pex19* mutant is lethal due to a deficit in medium-chain fatty acids (MCFAs). Increased lipolysis mediated by Lipase 3 (Lip3) leads to accumulation of free fatty acids and lipotoxicity. Administration of MCFAs prevents lipolysis and decreases the free fatty acid load. This drastically increases the survival rate of *Pex19* mutants without reducing VLCFA accumulation. We identified a mediator of MCFA-induced lipolysis repression, the ceramide synthase Schlank, which reacts to MCFA supplementation by increasing its repressive action on *lip3*. This shifts our understanding of the key defects in peroxisome-deficient cells away from elevated VLCFA levels toward elevated lipolysis and shows that loss of this important organelle can be compensated by a dietary adjustment.

## Author summary

Peroxisomes are organelles that contain several enzymes and fatty acids required for many metabolic tasks in the cell, and upon peroxisome loss, their educts accumulate. One example is the accumulation of very-long-chain fatty acids (VLCFAs) with a chain length of more than 20 carbons. These fatty acids cannot be oxidized in mitochondria but are exclusively degraded in peroxisomes. Lowering increased VLCFA levels is sometimes attempted as a treatment option for human disorders with peroxisomal dysfunction, although its effectiveness remains unclear. Here, we have analyzed this process in *Drosophila melanogaster* and found that peroxisomal loss results not only in VLCFA accumulation but also in a reduction of medium-chain fatty acids (MCFAs). We could show that this is due to a state of high lipolysis and increased mitochondrial activity. By supplementation with MCFAs from coconut oil, we were able to rescue mitochondrial damage and lethality observed in peroxisome-deficient flies. We found that this process is mediated by the ceramide synthase Schlank, which acts as a transcription factor and shuttles between nuclear membrane and endoplasmic reticulum (ER) in response to MCFA availability. We conclude that peroxisome loss triggers the accumulation of free fatty acids and mitochondrial damage in flies and that these effects can be rescued by a diet rich in MCFAs.

## Introduction

Peroxisomes are vesicular organelles originally discovered and described by C. De Duve as catalase-containing organelles important for the degradation of hydrogen peroxide [1]. Recently, it has become more and more apparent that they harbor much more complex metabolic functions, which are still incompletely understood. In mammalian cells, they are involved in the β-oxidation of very-long-chain fatty acids (VLCFAs), the formation of ether phospholipids (e.g., plasmalogens), the catabolism of branched-chain fatty acids, the production of bile acids, polyamine oxidation, and amino acid catabolism [2]. VLCFAs (chain length of C22 and more) do not enter the mitochondria via the carnitine shuttle carnitine palmitoyltransferase I (CPT I) and thus cannot be β-oxidized for energy gain. Instead, VLCFAs are exclusively oxidized in peroxisomes. They (and to a lesser extent, long-chain fatty acids [LCFAs], which are, however, predominantly oxidized in mitochondria) enter the peroxisomes after activation into acyl-CoA, where they are shortened by the peroxisomal β-oxidation machinery. The resulting short-chain fatty acids (SCFAs) and medium-chain fatty acids (MCFAs) are transported out of the peroxisome via the carnitine-shuttles carnitine O-acetyltransferase (CRAT) and carnitine O-octanoyltransferase (CROT) and enter the mitochondrion via carnitine shuttle or thiolase-dependent transport. In the mitochondria, they are further oxidized to acetyl-coA and feed the tricarboxylic acid (TCA) cycle and the electron transport chain [3]. It is unclear whether and to what extent the shortened peroxisomal products contribute to mitochondrial energy production, but overall, the energy gain from VLCFAs is minor in comparison to S/M/LCFAs because of their low abundance [4]. In yeast cells, peroxisomes are required for the β-oxidation of MCFAs [5], since there they are the only site of fatty acid oxidation.

The complex metabolic functions of peroxisomes are reflected by the multiform pathologic symptoms of peroxisomal biogenesis disorders (PBDs) of the Zellweger syndrome spectrum, whose study has greatly contributed to our understanding of peroxisomal function. PBDs are rare genetic diseases caused by mutations in one of the approximately 16 peroxin (PEX) genes, which are involved in the assembly and maintenance of peroxisomes. One major disease-causing effect is thought to be the accumulation of VLCFA-containing lipids, which occurs not only in PBDs but also in defects specific to the peroxisomal β-oxidation machinery, like X-linked adrenoleukodystrophy (X-ALD) or single enzyme defects, and is thought to be a result of abolished degradation of VLCFAs in peroxisomes. While accumulation of VLCFAs in these peroxisomal diseases is undisputed, there are still some doubts as to the exact underlying mechanism. Mice lacking a functional VLC-acyl-CoA synthetase, which is needed to activate VLCFAs in order to enter the peroxisome, show normal VLCFA levels, although peroxisomal β-oxidation is strongly reduced [6]. Similarly, the exact pathological effect downstream of accumulating VLCFAs remains elusive, although detrimental effects on mitochondria are postulated by many authors. Mitochondrial defects are indeed often observed in peroxisomal diseases [7–10], and in vitro assays show mitotoxic effects of VLCFAs added to cells [11,12]. However, the majority of accumulating VLCFAs in peroxisomal diseases is shown to be contained in lipids and not as free fatty acids [13], also called nonesterified fatty acids (NEFAs), in which form they were added to the cells. Furthermore, a number of authors observe normal mitochondrial function and morphology in VLCFA-enriched tissues carrying an X-ALD causing ATP-binding cassette family D transporter (ABCD1) mutation [14–16], which suggests other pathological mechanisms involved in mitochondrial dysfunction in PBDs.

The peroxisomal biogenesis and assembly machinery is well conserved in *Drosophila* [17–22]. In the present study, we use a *D*. *melanogaster Pex19* mutant [23] as a genetically tractable in vivo model system to elucidate the impact of peroxisomal deficiency on cellular and organismal metabolism. Pex19 is a predominantly cytoplasmic peroxisomal core factor and essential for both the import of peroxisomal membrane proteins (PMPs) and the de novo formation of peroxisomes [24,25]. Together with Pex3 and Pex16, it is responsible for the translocation of membrane proteins and membrane vesicle assembly [26]. Pex19 loss of function specifically leads to Zellweger syndrome, the severest form of PBDs. We have previously shown that major hallmarks of Zellweger syndrome are recapitulated in *Pex19* mutant flies, like absence of peroxisomes, VLCFA accumulation, mitochondrial abnormalities, and severely decreased viability. Furthermore, we have identified increased free fatty acids as a mitochondria-damaging agent in these flies. Free fatty acids accumulate as a result from a metabolic shift toward maximal lipid catabolism with severely increased lipolysis, which is caused by altered hepatocyte nuclear factor 4 (Hnf4) activity [23].

Here, we identify an imbalance in fatty acid composition in *Pex19* flies as a pathology-causing effect. While VLCFAs accumulate as expected in *Pex19* mutants, M/LCFAs are reduced to an extent far surpassing the relative effect caused by increased VLCFAs. This reduction is especially pronounced for fatty acids of 12 and 14 carbons (C12:0, C14:0, C14:1), and we here present evidence that this MCFA gap is involved in pathology progression in the absence of peroxisomes. Feeding a diet rich in C12- and C14-containing coconut oil (or other natural oils of similar composition) rescues *Pex19* flies into adulthood and ameliorates their metabolic imbalance, which is not achieved by feeding LCFA-containing oils. Similar rescues of *Pex2* and *Pex3* mutants unequivocally prove that peroxisomal absence, and not Pex19 loss itself, is responsible for the metabolic phenotype observed in *Pex19* mutants. We show that MCFA shortage results in a shift in subcellular localization of the ceramide synthase Schlank. Schlank was recently reported to harbor a secondary function as a transcription factor and was shown to repress *lipase 3* (*lip3*) [27]. Consistently, we observe severely increased expression of *lip3* expression in *Pex19* mutants. The feeding of coconut oil results in normalizing the activity of Schlank, concomitant with reduced *lip3* transcription, ultimately resulting in reduced lipolysis, reduced free fatty acid levels, and amelioration of the mitochondrial phenotype. Furthermore, we present evidence that this general pathological mechanism is also present in a Pex19-deficient patient cell line, thereby opening up a new path in the search for future therapies for PBDs and other peroxisomal diseases.

## Results

### Filling the MCFA gap rescues *Pex19* mutants

**Fig 1:**
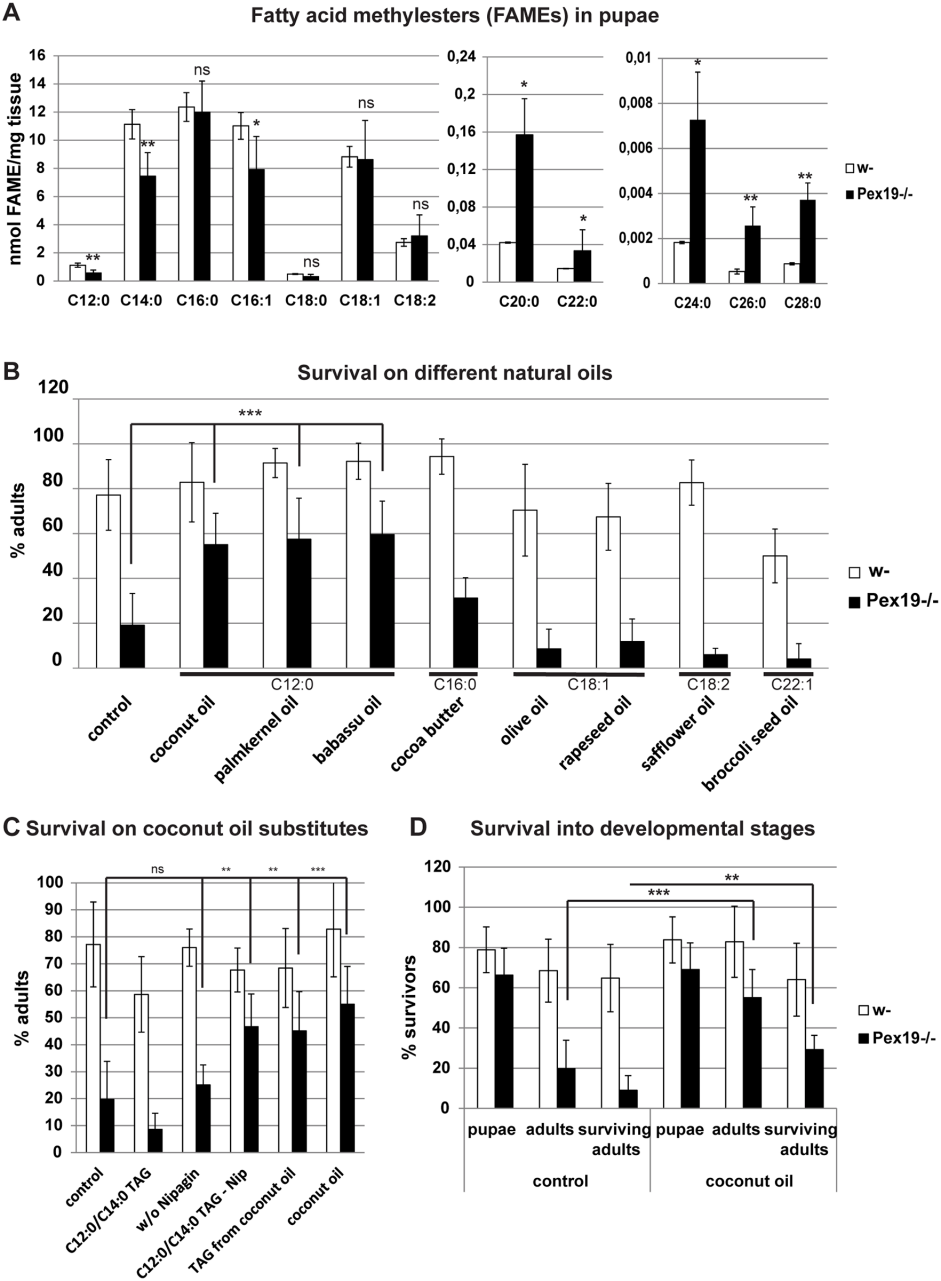
*Pex19* mutants display shortage in M/LCFAs and survive better when fed with MCFA-rich oils. (A) GC/MS analysis of FAMEs of wild-typic and *Pex19* mutant pupae, concentration in nmol/mg. *n* = 4. Significance tested with ANOVA. (B) Survival upon addition of different natural oils with different fatty acid compositions. *n* = 5 in groups of 25 individuals. (C) Survival upon addition of M/LC-TAGs. *n* = 5 in groups of 25 individuals. -Nip: without nipagin. (D) Survival profile with percentage of pupae, adults, and surviving adults upon rescue diet feeding. *n* = 10 in groups of 25 individuals. Significance tested using Student *t* test. Genotypes are w-: *w*^*1118*^, *Pex19−/−*: *w*^*1118*^; *Pex19*^*ΔF7*^*/Pex19*^*ΔF7*^. Error bars represent SD. *p < 0.05; **p < 0.01; ***p < 0.001. Corresponding raw data can be found in supplemental file S1 Data. FAME, fatty acid methyl ester; GC/MS, gas chromatography/mass spectometry; LCFA, long-chain fatty acid; MCFA, medium-chain fatty acid; TAG, triacylglycerol.

As we described previously, flies carrying a deletion of the *Pex19* gene locus (*Pex19*^*ΔF7*^, from here on referred to as *Pex19* mutants) lose their peroxisomes during larval development and die predominantly during the pupal stage [23]. When analyzing the fatty acid profile of *Pex19* mutant pupae, we found an unexpected shortage in M- and LCFAs in addition to the well-described accumulation of VLCFAs (SCFAs, MCFAs, LCFAs, and VLCFAs here refer to chain lengths of 4–8, 10–14, 16–18, and ≥20 C-atoms, respectively). Direct comparison of the absolute concentration of all fatty acid methyl esters (FAMEs) revealed a significant reduction of M/LCFAs, especially C12:0 and C14:0, while FAMEs of C20:0, C24:0, C26:0, and C28:0 are enriched (Fig 1A), which is consistent with our previous findings in larvae [23]. M/LCFAs can fuel mitochondrial ATP production and do not depend on chain shortening by the peroxisome before they can enter the mitochondrion and the TCA cycle. Since we suspected an energy deficit in *Pex19* mutants, we sought to fill the gap in MCFAs by dietary modifications. Freshly hatched larvae were kept by default on a high-caloric diet (apple juice agar plates with fresh yeast paste) rich in both carbohydrates and proteins. To avoid problems with toxicity or food avoidance, we used natural oils as sources for fatty acids with varying chain length. In order to minimize adverse effects of a high-fat diet [28], we added only 5% of the different natural oils to the yeast paste and analyzed *Pex19* mutant survival. We found that oils containing high amounts of lauric acid (C12:0) and myristic acid (C14:0)—like coconut oil, babassu oil, or palm kernel oil [29–31]—have a positive effect on the survival rate of *Pex19* mutants, yielding 55% of adult flies as compared to 20% on control food. By contrast, oils containing mainly LCFAs or VLCFAs have no or negative effects on the survival of *Pex19* mutants (Fig 1B). For further studies, we used coconut oil, which contains up to 60% lauric acid and up to 18% myristic acid [29,30]. To show that the fatty acid composition rather than vitamins or secondary plant compounds is responsible for the observed rescue effect, we fed purified triacylglycerols (TAGs) from coconut oil to *Pex19* mutants, which also resulted in a significant increase in adult flies (Fig 1C). Surprisingly, addition of synthetic TAGs containing only C12:0 or C14:0 does not copy the rescue effect of coconut oil, suggesting either impurities from the manufacturing process or other adverse effects of synthetic TAGs, since they slightly reduced the survival of control flies (Fig 1C). However, upon removal of the mitotoxic preservative nipagin from the food, synthetic TAGs also showed a rescue effect, while removal of nipagin alone does not enhance the survival of *Pex19* mutants (Fig 1C). Supplementation with 5% coconut oil does not only increase the number of adults hatching from the pupal case from 20% to 55% but also the number of adults that survive for more than 24 hours after hatching, from 9% to 29% (Fig 1D). These adult flies survived up to 3 weeks without further supplementation of coconut oil.

### MCFA rescue diet fills the MCFA gap without altering VLCFA levels

**Fig 2:**
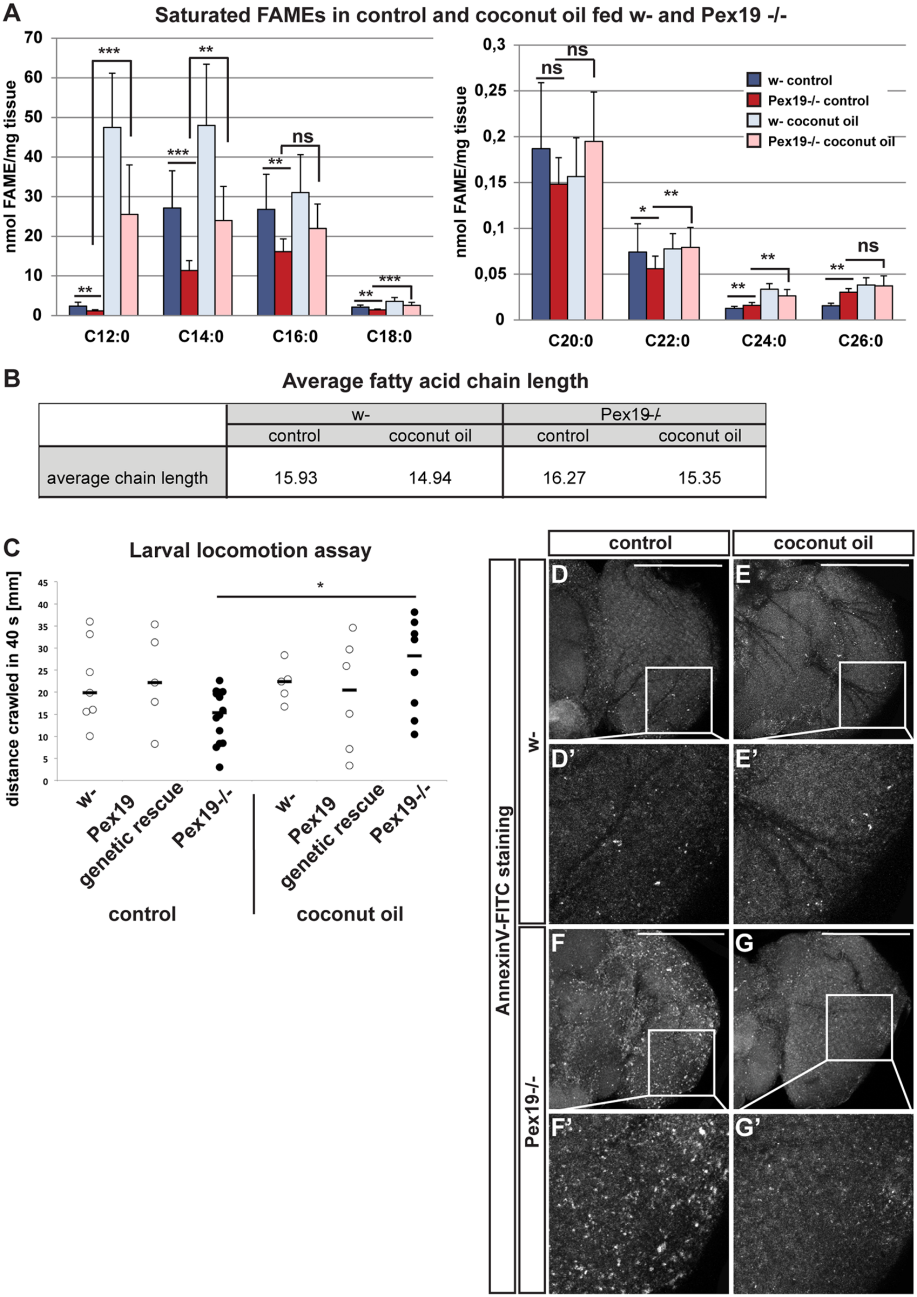
Effects of coconut oil rescue on *Pex19* mutants’ fatty acid profile and neurodegeneration. (A) FAMEs comparing w- and *Pex19−/−* fed with control and M/LCFA-enriched rescue food. Absolute concentration in nmol/mg wet tissue. *n* = 7. Significance tested with ANOVA. (B) Calculation of the average chain length from the absolute FAME values. (C) Larval locomotion assay as a readout for neurodegeneration. Dots represent single experiments, bars represent median. Significance tested with Student *t* test. (D-G) Annexin V-FITC staining of 1-day-old adult brains. Scale bars represent 100 μm. *n* = 5. Error bars represent SD. *p < 0.05; **p < 0.01; ***p < 0.001. Corresponding raw data can be found in supplemental file S1 Data. FAME, fatty acid methyl ester; FITC, fluorescein isothiocyanate; M/LCFA, medium- and long-chain fatty acid; ns, not significant.

The inability to break down VLCFAs is considered the main disease-mediating factor in cells without functional peroxisomes, and lowering VLCFA levels is the aim of most therapies for patients with defective peroxisome metabolism, mostly by inhibiting elongases, e.g., with erucic acid–containing Lorenzo’s oil [32,33]. We asked whether the dietary administration of 5% coconut oil had a rescue effect by lowering the VLCFA content and reanalyzed FAMEs in coconut oil–fed larvae by gas chromatography/mass spectrometry (GC/MS). We found that the MCFA-enriched diet leads to an increase in lipids containing C12:0 and C14:0 in both control and *Pex19* mutant animals, both absolutely and relatively (Fig 2A, supplemental file S1 Fig). The total amount of fatty acids as a measure for lipids is reduced in *Pex19* mutants, while, upon coconut oil administration, it is elevated in both wild types and *Pex19* mutants (supplemental file S1 Fig). The relative amounts of lipid-contained fatty acids with a chain length of C16:0 are reduced, despite the fact that coconut oil contains up to 9% of C16:0, which suggests that the fatty acids from the rescue diet alter the FAME profile instead of simply representing the additional fatty acids taken up from the M/LCFA-enriched diet (supplemental file S1 Fig). Unexpectedly, lipids containing VLCFAs from C22:0 to C26:0 remain at elevated levels in *Pex19* mutants and even increase in control animals on coconut oil–supplemented food (Fig 2A, supplemental file S1 Fig). Despite this increase, coconut oil supplementation has a beneficial effect on the survival of wild-typic flies as well as *Pex19* mutants (Fig 1D). We calculated the average chain length from the absolute concentration of FAMEs and found that administration of the MCFA-enriched diet reduces the chain length from an average of 15.93 to 14.94 carbons in wild types and from an average of 16.27 to 15.35 carbons in *Pex19* mutants (Fig 2B), showing that food-derived fatty acids have an influence on overall fatty acid content in flies, consistent with overall changes in the lipidome according to food source [34].

### MCFA rescue diet prevents neurodegeneration

Adult *Pex19* mutants appeared weak and did not properly inflate their wings. We tested their climbing ability in a negative geotaxis assay as a readout for neurodegeneration and found that *Pex19* mutants climb slower than wild-typic flies and that most of them do not climb at all. Upon coconut oil supplementation, the climbing speed of *Pex19* mutants increases, and the number of flies which are not able to climb decreases (supplemental file S1 Fig). Since we were limited in the number of adults because of the early lethality of the *Pex19* mutants, we also conducted a crawling assay as a readout for larval neurodegeneration. We found that w- controls and *Pex19* mutants with a genetic rescue construct show similar crawling performance on control and coconut oil–supplemented food, whereas *Pex19* mutant larvae have locomotion deficits. The low crawling performance of *Pex19* mutants significantly improves upon coconut oil supplementation (Fig 2C). To further characterize neurodegeneration in *Pex19* mutants, we analyzed apoptosis in brains of 1-day-old adults by staining them with Annexin V-FITC (fluorescein isothiocyanate) (Fig 2D–2G). We found that *Pex19* mutants have higher numbers of apoptotic cells in the brain. Supplementation with coconut oil results in a marked decrease of apoptotic cells in *Pex19* mutants (Fig 2F and 2G). This shows that neurodegeneration due to peroxisome loss can be prevented or slowed down by dietary administration of MCFAs.

### MCFA rescue diet ameliorates mitochondrial phenotypes

**Fig 3:**
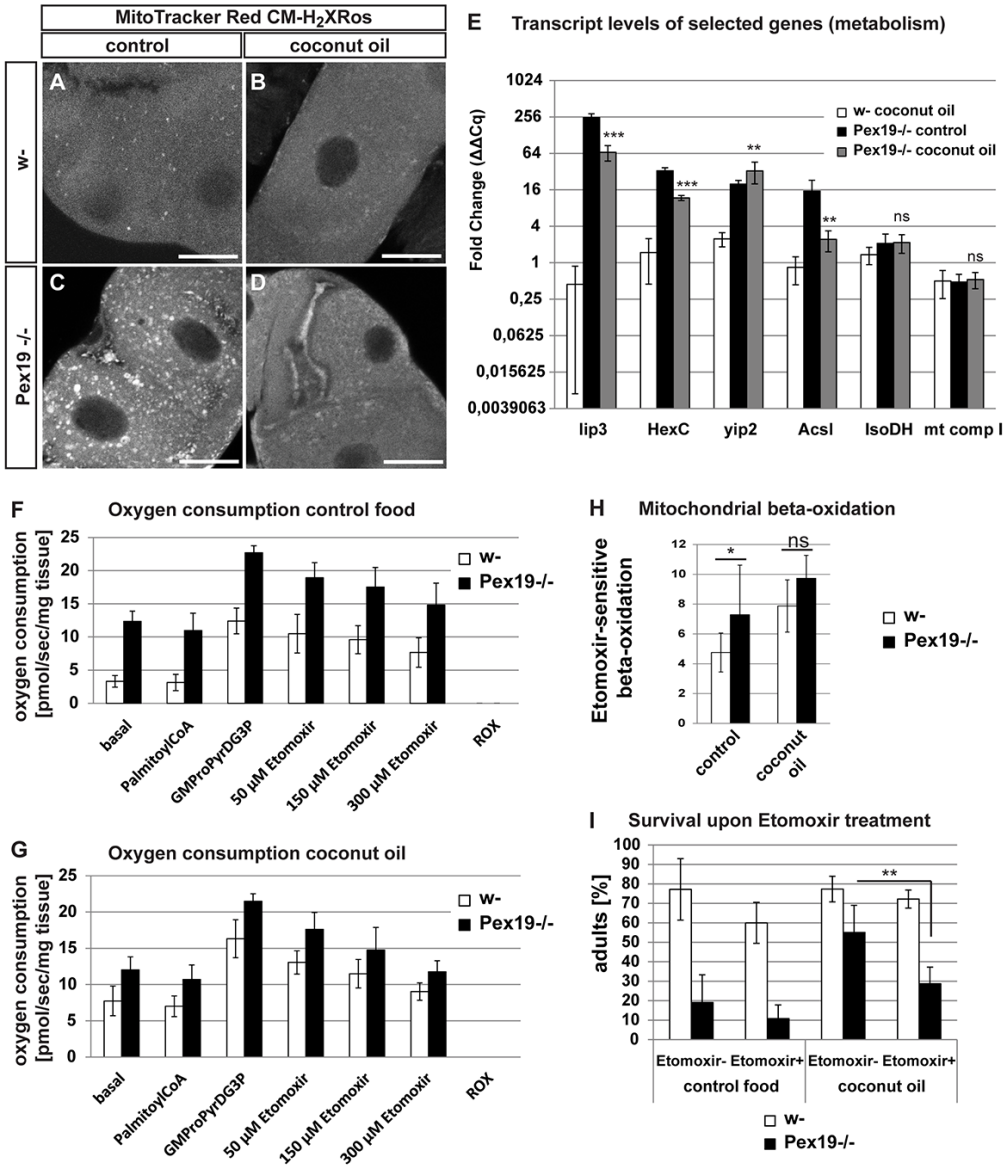
Effects of dietary administration of M/LCFA on mitochondria and metabolism. (A-D) Staining of mitochondria with MitoTracker Red CM-H2XRos to show production of ROS. Scale bars represent 10 μm. *n* = 5. (E) Real-time qPCR analysis of genes encoding for metabolic enzymes. ΔCq values are normalized to w- (ΔΔC_q_ or fold regulation). (F, G) Oxygen consumption levels of wild-type and *Pex19* mutant larvae (G: glutamate, M: malate, Pro: proline, Pyr: pyruvate, D: ADP, G3P: glycerol 3 phosphate). *n* = 4. (H) Mitochondrial β-oxidation rate under control and rescue condition. (I) Addition of 25 μM etomoxir reduces the survival rate of coconut oil–fed *Pex19−/−*. *n* = 10 in groups of 25 individuals. Genotypes are w-: *w*^*1118*^, *Pex19−/−*: *w; Pex19*^*ΔF7*^*/Pex19*^*ΔF7*^. Error bars represent SD. *p < 0.05; **p < 0.01; ***p < 0.001. Significance tested with Student *t* test. Corresponding raw data can be found in supplemental file S1 Data. Acsl, acyl-CoA synthetase long-chain; HexC, hexokinase C; IsoDH, isocitrate dehydrogenase; lip3, lipase 3; M/LCFA, medium- and long-chain fatty acid; mt comp I, mitochondrial complex I; qPCR, quantitative PCR; ROS, reactive oxygen species; ROX, residual oxygen consumption; yip2, yippee-interacting protein.

Peroxisome loss provokes mitochondrial swelling due to elevated lipolysis and free fatty acid levels, as was shown by tetramethylrhodamine ethyl ester (TMRE) and mitotracker green stainings, as well as on the ultrastructural level (transmission electron microscopy, TEM) [23]. In order to assess the impact of the MCFA rescue diet on mitochondria, we stained mitochondria in the Malpighian tubules of third-instar larvae with MitoTracker Red CM-H_2_XRos. This dye emits fluorescence after oxidation in the cell and thus detects reactive oxygen species (ROS). We found that tissue of *Pex19* mutants contains high amounts of ROS, as indicated by high numbers of enlarged CM-H_2_XRos-positive mitochondria. Upon coconut oil administration, size and number of CM-H_2_XRos-positive mitochondria decrease (Fig 3A–3D).

### The rescue diet normalizes transcript levels of *lip3* and other metabolic enzymes

To investigate the impact of the MCFA diet on the metabolism of *Pex19* mutant animals, we analyzed several genes encoding for metabolic enzymes (Fig 3E). We found that expression of the acid lipase *lip3*, which is highly up-regulated in *Pex19* mutants, is reduced from 250-fold to 66-fold upon feeding of the rescue diet. Previously, we could show that *lip3* expression correlates with free fatty acid accumulation [23].

Hnf4 target genes such as *hexokinase C* (*HexC*), *yippee-interacting protein 2* (*yip2*), and *acyl-CoA synthetase long-chain (Acsl*) are up-regulated in *Pex19* mutants [23]. Upon coconut oil supplementation, expression of the glycolysis enzyme *HexC* is reduced from 33-fold to 11-fold, and the acyl-CoA synthetase Acsl is reduced from 15-fold to 2-fold (Fig 3E). By contrast, the acetyl-CoA acyltransferase *yip2* remains at high levels on rescue food, suggesting that mitochondrial β-oxidation stays at a maximum in *Pex19* mutants on MCFA rescue food and that the transcriptional response upon coconut oil rescue is markedly different than upon genetic reduction of Hnf4 with reduced *yip2* expression [23]. *Isocitrate dehydrogenase* (*IsoDH*), a target gene of the regulator of mitochondrial abundance and peroxisome proliferator–activated receptor gamma coactivator 1-α (PGC1-α) ortholog *spargel* [35], is slightly up-regulated in *Pex19* mutants and remains at this level upon feeding of the rescue diet, while another spargel target gene, *mt Complex I*, is down-regulated and also remains at this level upon treatment with coconut oil (Fig 3E).

### MCFAs from the rescue diet act on mitochondrial metabolism

*Pex19* mutants have increased β-oxidation rates, probably in response to the high free fatty acid load, and presumably without gaining additional energy from it (Fig 3F and 3H). We measured the mitochondrial β-oxidation in wild-type and *Pex19* mutants fed with coconut oil as the etomoxir-sensitive oxygen consumption of permeabilized larval tissue. Amounts of porin and citrate synthase activity were determined to exclude effects due to higher mitochondrial mass (supplemental S2A and S2B Fig). We found that the rescue diet enhances the β-oxidation rate in wild types and found that it stays at high levels in *Pex19* mutants under both feeding conditions. This suggests that MCFAs increase mitochondrial β-oxidation in general while possibly improving the energy gain in *Pex19* mutants, since they already have maximal β-oxidation rates under normal food conditions (Fig 3G and 3H).

Since mitochondrial β-oxidation is increased in *Pex19* mutants, the question arises if the gap in MCFAs and the rescue effect of MCFA-containing coconut oil is caused by their preferential degradation in mitochondrial β-oxidation for energy gain. To show that the MCFAs from coconut oil target the mitochondria, we cotreated the larvae with the CPT I–inhibitor etomoxir [36]. Etomoxir indeed abolishes the rescue effect of coconut oil with respect to survival (Fig 3I). This suggests that MCFAs from the rescue diet are efficiently metabolized by the mitochondria.

### Effect of the rescue diet to other Pex mutants

**Fig 4:**
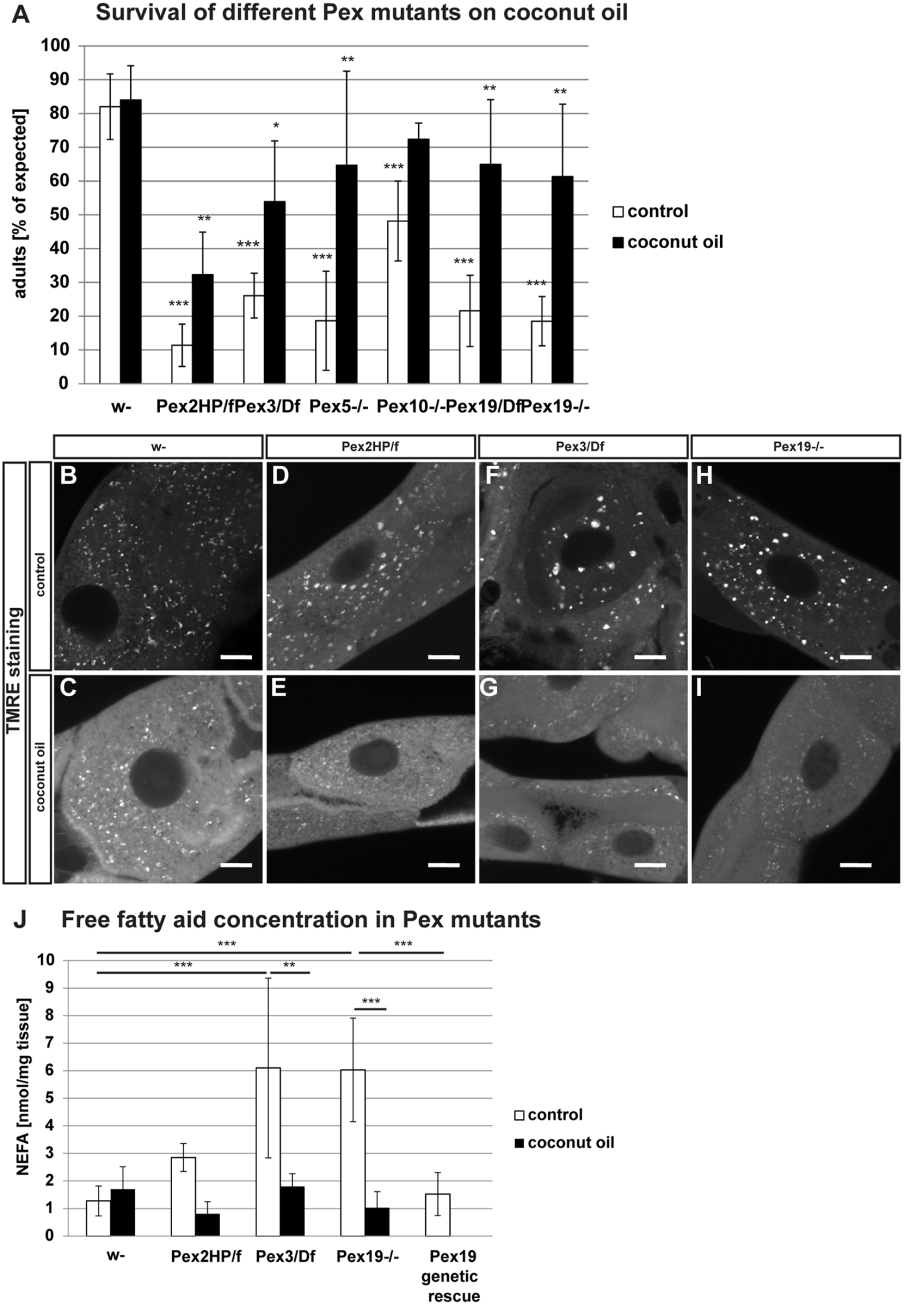
Other *Pex* mutants show a phenotype similar to *Pex19* mutants and can also be rescued with coconut oil. (A) Survival of different *Pex* mutants on control and rescue diet. *n* = 5 in groups of 25 individuals. (B-I) TMRE staining of third-instar larval Malpighian tubules. Scale bars represent 10 μm. *n* = 5. (J) FAMEs comparing w- and Pex mutants fed with control and M/LCFA-enriched rescue food. *n* = 3. Genotypes are *Pex2*^*HP35039*^*/Pex2*^*f01899*^, *Pex3*^*2*^*/ Df(32)6262*, *Pex5*^*MI06050*^*/ Pex5*^*MI06050*^, *Pex10*^*MI04076*^*/ Pex10*^*MI04076*^, *Pex19*^*ΔF7*^*/ Df(2L)esc* ^*p3-0*^, *Pex19*^*ΔF7*^*/ Pex19*^*ΔF7*^. Error bars represent SD. Asterisks represent *p < 0.05, **p < 0.01, ***p < 0.001. Significance tested using ANOVA with Tukey posttest. Corresponding raw data can be found in supplemental file S1 Data. FAME, fatty acid methyl ester; M/LCFA, medium- and long-chain fatty acid; NEFA, nonesterified fatty acid; Pex, peroxin; TMRE, tetramethylrhodamine ethyl ester.

The striking rescue of the loss of a whole organelle by a dietary adjustment provoked the question whether it was specific for *Pex19* mutants or general for the loss of peroxisomes. We tested the effect of the dietary rescue on the survival of other Pex mutants: transheterozygous *Pex2*^*HP35039*^*/Pex2*^*f01899*^ [37], referred to as *Pex2*^*HP/f*^; *Pex3*^*2*^[19] crossed over a deficiency (Df(32)6262), referred to as *Pex3/Df*; *Pex5*^*MI06050*^ (Bloomington stock collection), referred to as *Pex5*−/−; and *Pex10*^*MI04076*^ (Bloomington stock collection), referred to as *Pex10−/−*. Of note, it has been shown that *Pex2* and *Pex16* mutants have reduced levels of C12:0 [22], consistent with our observations in *Pex19* mutants. We also reanalyzed the survival and dietary rescue of the *Pex19*^*ΔF7*^ mutant allele crossed over a deficiency (*Df(2L)esc* ^*p3-0*^). We found that addition of 5% coconut oil to the food improves the survival of all of these Pex mutants (Fig 4A). To exclude unspecific effects due to enhanced food uptake, we performed a feeding assay but found no significant differences between genotypes or feeding condition (supplemental file S2 Fig). We concluded that the dietary rescue compensates for the loss of peroxisomes rather than for the loss of Pex19.

### MCFA diet ameliorates mitochondrial swelling in Pex mutants

To strengthen the hypothesis that the dietary rescue with coconut oil supplementation is a peroxisome rather than a Pex19-specific effect, we analyzed mitochondrial swelling in Malpighian tubules of *Pex2*^*HP/f*^, *Pex3/Df*, and *Pex19* mutant larvae. Similar to *Pex19* mutants, *Pex2* and *Pex3* mutants display large, balloon-shaped mitochondria, whereas mitochondria are small in wild-typic tissue. Upon administration of 5% coconut oil with the diet, we observed a rescue of this phenotype in all mutants: mitochondria were small and numerous, similar to wild-typic mitochondria (Fig 4B–4I). Similarly, the concentration of lipotoxic free fatty acids, which can cause mitochondrial swelling [23], is elevated in *Pex2* and *Pex3* mutants as in *Pex19* mutants. Feeding of the rescue diet reduces the free fatty acid concentration in all three Pex mutants. A *Pex19* mutant with a genetic rescue construct does not show elevated free fatty acid levels (Fig 4J).

### MCFA diet restores Schlank nuclear localization

**Fig 5:**
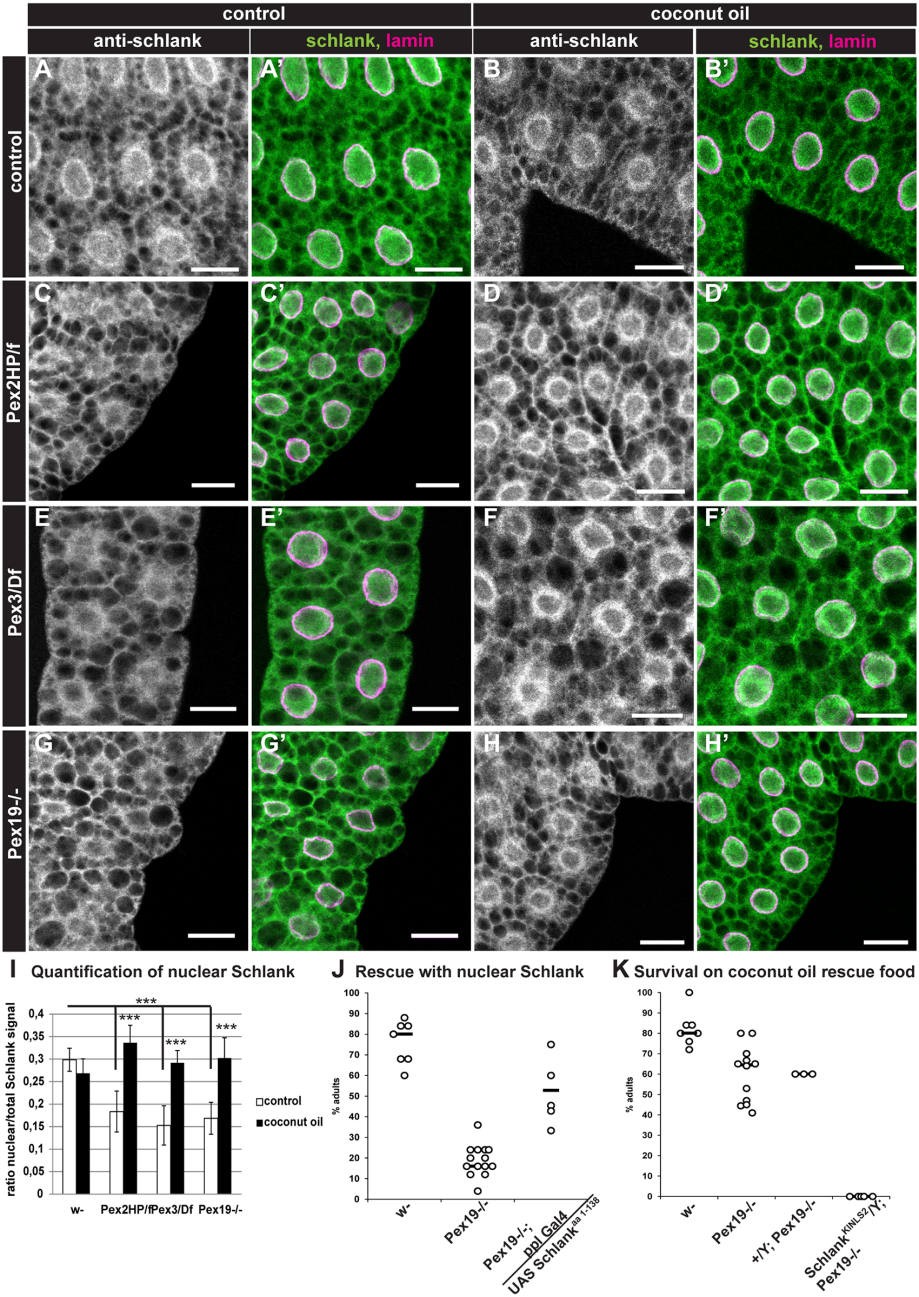
The dietary rescue functions by restoring Schlank nuclear localization. (A-H) Staining of larval fat body tissue with α-Schlank and α-Lamin. Scale bars represent 20 μm. (I) Quantification of the Schlank nuclear fluorescent signal. Bars represent the ratio between CTCF from nuclear region (within Lamin staining) and whole cell. (J) Constitutively nuclear Schlank^aa1–138^ rescues *Pex19* mutants. Genotypes are *w-*, *Pex19*^*ΔF7*^*/Pex19*^*ΔF7*^, *Pex19*^*ΔF7*^*UAS Schlank*^*aa1–138*^*/Pex19*^*ΔF7*^; *ppl-Gal4/+*. (K) *Pex19 Schlank KINLS2* double mutants are not rescued by the M/LCFA-enriched diet. Genotypes are *w-*, *Pex19*^*ΔF7*^*/Pex19*^*ΔF7*^, *+/Y; Pex19*^*ΔF7*^*/Pex19*^*ΔF7*^, *Schlank*^*KINLS2*^*/Y; Pex19*^*ΔF7*^*/Pex19*^*ΔF7*^. *n* ≥ 3 in groups of 25 individuals. Error bars represent SD. Dots represent single experiments. Black bars represent median. *p > 0.05, ***p < 0.001. Significance tested using ANOVA with Tukey posttest. Corresponding raw data can be found in supplemental file S1 Data. aa, amino acid; CTCF, corrected total cell fluorescence; KINLS2, knock-in nuclear localization sequence 2; M/LCFA, medium- and long-chain fatty acid; UAS, upstream activating sequence.

*Lip3* expression is drastically increased in *Pex19* mutants [23], which is reduced upon feeding of coconut oil (Fig 3E). Recently, it was discovered that the ceramide synthase Schlank acts as a transcriptional regulator of *lip3* [27]. Schlank shuttles from the nuclear envelope to the endoplasmic reticulum (ER) membrane, thereby releasing its repression of *lip3* transcription. We assessed the subcellular localization of Schlank in *Pex2*, *Pex3*, and *Pex19* mutant fat body tissue by immunohistochemical stainings with anti-Schlank and anti-Lamin to stain the nuclear membrane. We found that the nuclear localization is indeed reduced (Fig 5C, 5E, 5G and 5I). This is consistent with the observed elevated transcript levels of *lip3* and free fatty acid levels.

We wanted to know if Schlank plays a role in the rescue effect induced by MCFA supplementation. Of note, upon addition of 5% coconut oil to the diet, Schlank indeed regained its localization to the nuclear membrane (Fig 5D, 5F, 5H and 5I) in the *Pex2*, *Pex3*, and *Pex19* mutants we analyzed and would thus be enabled to repress *lip3*, explaining the observed decrease in *lip3* expression, reduced free fatty acid levels, ameliorated mitochondrial morphology, and rescue to adulthood. To further prove this hypothesis, we overexpressed a shortened version of Schlank (Schlank^aa1–138^) [38,27], which is constitutively nuclear (supplemental file S3 Fig), in the *Pex19* mutant background. We found that forcing Schlank into the nucleus in this manner rescues the lethality of *Pex19* mutants (Fig 5J). Furthermore, we generated a double mutant for *Pex19* and a *Schlank knock-in* (KI) mutant with a mutation in one of the nuclear localization sequences (NLS2), *Schlank*^*KINLS2*^ [28,27]. *Schlank*^*KINLS2*^ mutants show derepression of *lip3*, since the mutant Schlank protein is excluded from the nuclear membrane [27]. Without the ability to enter the nucleus, Schlank^KINLS2^ protein should not be able to react to MCFA supplementation, and a rescue with coconut oil should no longer be possible. Indeed, we found that *Schlank*^*KINLS2*^; *Pex19* double mutants are larval lethal and are not rescued by coconut oil (Fig 5K), which confirms a role of Schlank in conferring the rescue effect of MCFA supplementation: Schlank releases its repression on *lip3* due to MCFA shortage in *Pex19* mutants, thereby provoking lipotoxicity and increased mitotoxic free fatty acid levels. Filling the MCFA gap via the diet restores Schlank nuclear localization and thus its repression of *lip3*, which ameliorates the damaging lipolytic program. If Schlank lacks the nuclear localization sequence, the dietary rescue no longer works, since the mutated Schlank cannot confer *lip3* repression by shuttling to the nuclear membrane.

### MCFAs rescue mitochondrial damage in PEX19-deficient human fibroblasts

We posed the question whether rescue of peroxisome loss was specific for *D*. *melanogaster* or a more universal effect. To address this question, we made use of two other models: *Caenorhabditis elegans* and, to assess the clinical relevance of our findings, a human skin fibroblast cell line from a Zellweger syndrome patient with a mutation in PEX19 (Δ19T cells, [39], supplemental file S3 Fig).

We fed a *C*. *elegans* strain expressing mitochondrial GFP in muscle tissue (*myo-3p*::*mtGFP)* with bacteria expressing RNA interference (RNAi) against *prx-19*, the *C*. *elegans* Pex19 homolog. Knock-down of *prx-19* led to massively swollen mitochondria. When coconut oil was added to the bacterial lawn, control worms showed some fragmented and enlarged mitochondria, while mitochondrial swelling in the *prx-19* knock-down animals was reduced (supplemental file S3 Fig).

**Fig 6:**
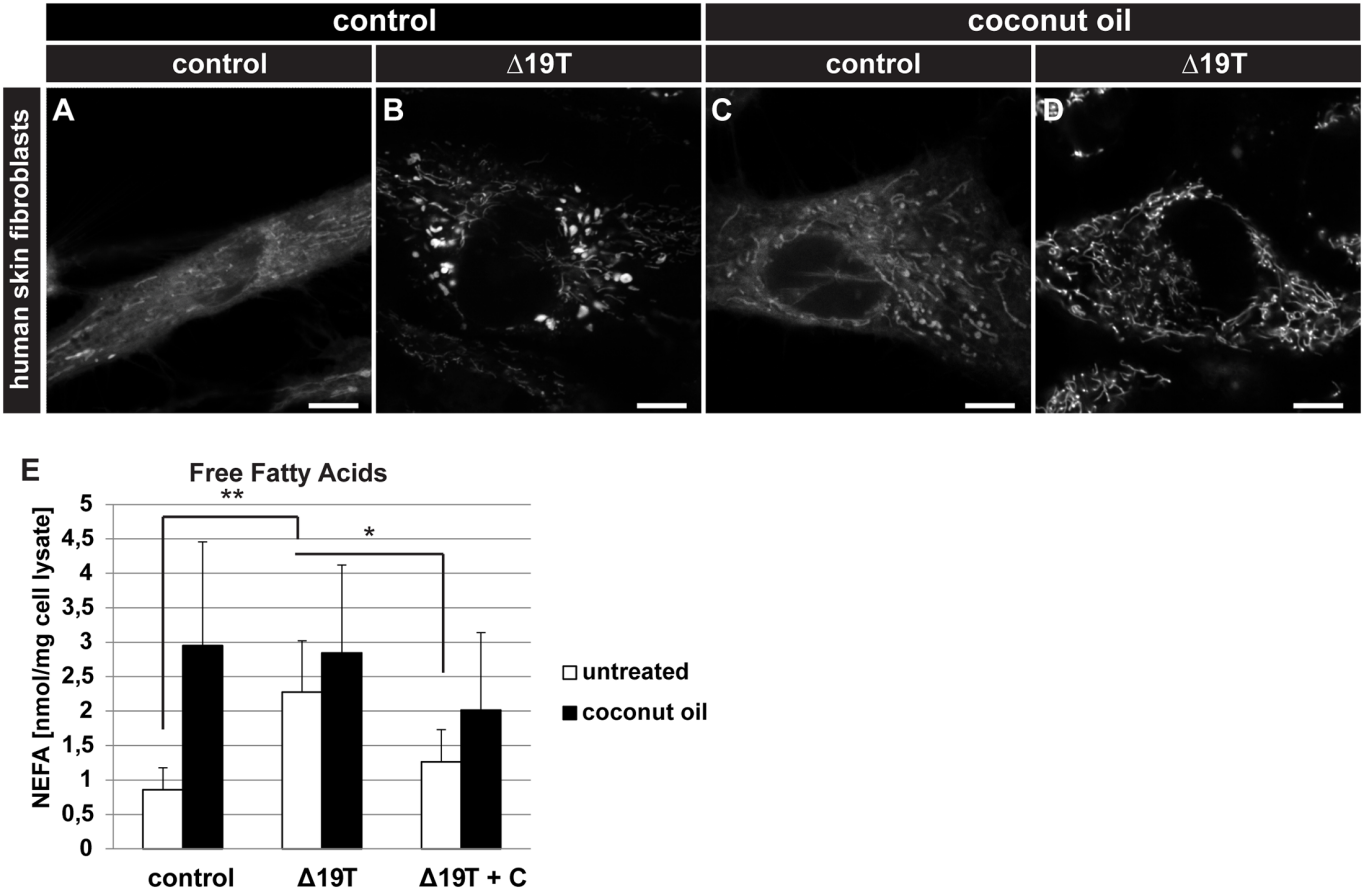
Conservation of the dietary rescue in human fibroblasts. (A-D) TMRE staining of human skin fibroblasts with a PEX19 mutation (Δ19T). Comparison of untreated and BSA-conjugated coconut oil–treated cells. Scale bars represent 10 μm. *n* = 10. (E) Concentration of free fatty acids in human skin fibroblast cell lysates in untreated and BSA-conjugated coconut oil–treated cells. In addition, Δ19T were cultured in the presence of BSA-conjugated coconut oil (+C), and samples were taken in the absence of coconut oil–BSA. *n* = 5. Error bars represent SD. * *p* < 0.05, ** *p* < 0.01. Significance tested using Student *t* test. Corresponding raw data can be found in supplemental file S1 Data. BSA, bovine serum albumine; NEFA, nonesterified fatty acid; PEX, peroxin; TMRE, tetramethylrhodamine ethyl ester.

We analyzed the mitochondrial morphology in control fibroblasts from a healthy person and in Δ19T cells by staining them with TMRE and found that mitochondria were organized in long, stretched, filamentous networks in control cells, while they were more fragmented and swollen in Δ19T cells. To mimic the M/LCFA rescue diet, we conjugated coconut oil to bovine serum albumin (BSA, final concentration approximately 1 mM) and added 10% to the media. Similar to our results in *C*. *elegans*, this leads to mitochondrial fragmentation in control cells but has an ameliorative effect on the mitochondrial swelling in Δ19T cells (Fig 6A–6D, supplemental file S3 Fig).

Free fatty acid levels are elevated in Δ19T cells (Fig 6E). Upon addition of 10% BSA-conjugated coconut oil, free fatty acid levels increased in both control and Δ19T cells. By contrast, when Δ19T fibroblasts were cultured with 10% BSA-conjugated coconut oil for several passages, and samples were taken in the absence of coconut oil in the medium, they showed reduced levels of free fatty acids (Fig 6E). Our results suggest a conserved mechanism of the impact of *Pex19* mutation on mitochondria and the pathology of the disease, converging on free fatty acid toxicity. Free fatty acid levels and mitochondrial swelling are ameliorated across vertebrate and invertebrate models (flies and human cells) upon supplementation with MCFA from coconut oil.

## Discussion

Accumulation of VLCFAs is thought to be one of the main pathology-driving effects of peroxisomal loss, and many authors claim mitotoxic effects of VLCFAs and phytanic acid, a branched-chain fatty acid accumulating in PBDs (for a recent review: [16]). On the other hand, there are a number of contradictory studies observing normal mitochondrial function and morphology in VLCFA-enriched tissues [14,15], which suggests other, so-far unidentified pathological mechanisms. Here, we provide evidence that in a *Drosophila* PBD model, mitotoxic effects of peroxisomal loss can be ameliorated without altering overall VLCFA levels by providing MCFAs, which are depleted in *Pex19* mutants. The central finding of our study is the observation that MCFA shortage plays a more important role in the pathophysiology of peroxisome loss than VLCFA accumulation. MCFA shortage correlates with free fatty acid accumulation and subsequent mitochondrial damage, which can be overcome by administering MCFA-rich coconut oil. Importantly, this measure does not reduce VLCFA levels in *Pex19* mutants and even increases VLCFA levels in the wild type, although mitochondrial pathology is clearly improved and neurodegeneration and lethality counteracted. While VLCFAs are obviously mitotoxic in micromolar concentrations when administered to cells or tissue as free fatty acids [11,12,16], we argue that their accumulation in vivo is, while certainly not without adverse consequence for the organism, less severe due to their main presence in lipids instead of in their nonesterified form. Furthermore, we measure VLCFA concentrations in the nanomolar, not micromolar, range. Instead, we observe increased levels of free fatty acids as the mitotoxic agent, which accumulate as the result of dysregulated lipolysis.

Free fatty acids are toxic for the cell; therefore, lipolysis and degradation of fatty acids is tightly coregulated to keep the overall level of free fatty acids low and avoid lipotoxic effects on mitochondria. This is achieved by their role as activating ligands for Hnf4 in *Drosophila* (peroxisome proliferator–activated receptors [PPARs] in vertebrates) [40], which in response induces a lipid catabolic program. In the absence of peroxisomes, we observe that this program is “maxed out” and produces more fatty acids than the mitochondria can handle (as indicated by maximal mitochondrial β-oxidation in *Pex19* mutants, even when fed the rescue diet), resulting in mitochondrial damage and ultimately energy deficit and death. It was shown that peroxisomes can protect mitochondria from fatty acid overload in the myocardium after ischemia-reperfusion injury: if mitochondrial β-oxidation is reduced, peroxisomal β-oxidation increases, resulting in lowering overall free fatty acid levels and protection of the myocardial cells [41], which is consistent with a general role of peroxisomes in regulating overall free fatty acid levels and mitoprotection.

**Fig 7:**
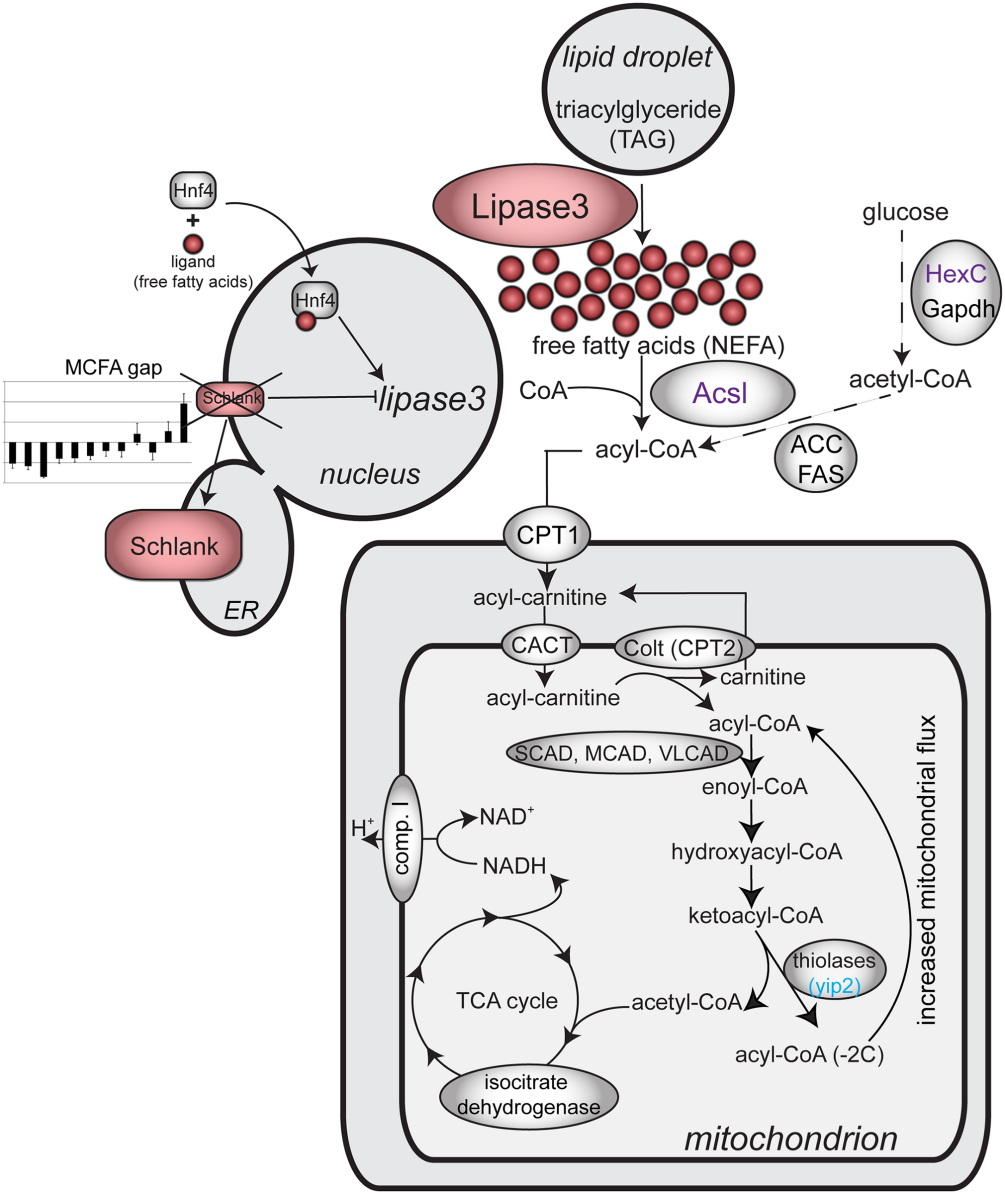
Summary of the role of Schlank in the pathology of *Pex19* mutants. The ceramide synthase Schlank leaves the nuclear membrane compartment in *Pex19* mutants as a result of reduced amounts of MCFA, thereby releasing repression of *lip3*. As a result, increased lipolytic activity of Lip3 leads to release of free NEFAs from fat stores. In a previous study, we identified Hnf4 as a regulator of increased lipolysis in *Pex19* mutants: upon fatty acid binding, Hnf4 enters the nucleus and activates target genes, among them *lip3*, thereby initiating a spiral of lipolysis and further Hnf4 activation by NEFA. Upon coconut oil feeding, the MCFA gap is filled, and Schlank reenters the nuclear membrane compartment and regains its ability to repress *lip3*, thereby ameliorating the *Pex19* phenotype and reducing the free fatty acid load. Transcriptional analysis revealed that the Hnf4 target genes *HexC* and *Acsl* are normalized upon coconut oil treatment (purple), while the opposite is true for another Hnf4 target: *yip2* is even more highly expressed on the coconut oil diet (blue). ACC, acetyl-CoA carboxylase; *Acsl*, acyl-CoA synthetase long-chain; CACT, carnitine acylcarnitine translocase; Colt, congested-like trachea; CPT, carnitine palmitoyltransferase; ER, endoplasmic reticulum; FAS, fatty acid synthase; GAPDH, glyceraldehyde 3-phosphate dehydrogenase; *HexC*, hexokinase C; Hnf4, hepatocyte nuclear factor 4; Lip3, lipase 3; MCAD, medium-chain acyl-CoA dehydrogenase; MCFA, medium-chain fatty acid; NEFA, nonesterified fatty acid; SCAD, short-chain acyl-CoA dehydrogenase; TCA, tricarboxylic acid; VLCAD, very-long-chain acyl-CoA dehydrogenase; yip2, yippee-interacting protein 2.

We unravel a mechanism by which MCFA shortage mediates Schlank nuclear exclusion, allowing for high expression of *lip3*, thereby inducing the production of free fatty acids. In a previous study, we identified Hnf4 as the central regulator of the resulting lipid catabolic program: the increase in free fatty acid levels caused by *lip3* up-regulation induces a spiral of hyperactive Hnf4 signaling, more lipolysis, and mitochondrial swelling [23]. We here provide evidence that the initial insult and entry point into the spiral is misregulation of *lip3* expression, caused by exclusion of Schlank from the nuclear membrane in *Pex19* mutants. As a result, *lip3* is derepressed and produces increased amounts of free fatty acids, thereby activating Hnf4 and the pathological cascade (summarized in Fig 7).

Strikingly, MCFA supplementation ameliorates lipotoxicity by restoring *lip3* repression by Schlank, while VLCFA levels remain high. We therefore conclude that reduced amounts of MCFA cause Schlank to leave the nucleus and stop its repressive action on *lip3*.

Interestingly, a recent study could show that the zebrafish Schlank homologue ceramide synthase 2 reacts to increased levels of sphingosine by enriching in the nuclear membrane compartment, similar to Schlank in response to coconut oil supplementation [42]. The enzymes responsible for producing the sphingosine precursor sphinganine are the serine palmytoyltransferases Spt1 and Spt2 (the latter is known as Lace in *Drosophila*). Of note, the educts for the condensation reaction catalyzed by Lace are serine and the MCFA lauric acid, which is the main fatty acid present in coconut oil (for a review on *Drosophila* sphingolipid metabolism, see [43]). The medium-chain sphinganine is then further oxidized to yield sphingosine, which is an educt for the acylation reaction catalyzed by Schlank to produce ceramide. It is therefore conceivable that providing lauric acid from coconut oil rescues *Pex19* mutants by feeding into sphinganine/sphingosine production, which allows Schlank to enrich in the nuclear membrane and repress *lip3* expression, thereby reducing lipotoxicity and lethality of *Pex19* mutants.

We show that mitochondrial damage, lipotoxicity, and Schlank mislocalization contribute substantially to pathophysiological defects not only in *Pex19* mutants but also in flies deficient in *Pex2* and *Pex3*, and that principle aspects of this cascade are conserved in human cells.

While we do not rule out that accumulation of VLCFA contributes to phenotype progression in PBDs, we identify a different lethality-inducing cascade driven by Hnf4 and Schlank that results in free fatty acid overload and mitotoxicity, independent of VLCFA levels. Our findings highlight that the results of peroxisomal dysfunction lead to more complex metabolic shifts than just the accumulation of educts and absence of products and argue for a central role of peroxisomes in balancing the lipid metabolism of an organism. Earlier studies observed that peroxisomal β-oxidation rates are influenced by mitochondrial β-oxidation rates and that VLCFA levels are, to a degree, independent of peroxisomal β-oxidation [14]. Our data not only show that the opposite is also true (peroxisomal loss influences mitochondrial β-oxidation), but we furthermore identify the ceramide synthase Schlank as a mediator of these metabolic changes.

Taken together, we show that shortage in MCFAs upon peroxisome loss induces a lipolytic program, leading to free fatty acid accumulation and mitochondrial damage, and that these effects can be counteracted by a dietary adjustment and are conferred by the ceramide synthase Schlank.

### Conclusion

We show that supplementation with MCFAs, rather than removal of VLCFAs, rescues a peroxisome-deficient PBD model by acting on the ceramide synthase Schlank to prevent lipotoxicity.

## Methods

### Fly husbandry

The *Pex19* mutant was generated by imprecise excision following *Drosophila*-standard techniques. The line *Pex19*^*ΔF7*^ was chosen from a jump-out screen and tested as a transcript null. To detect homozygous animals, they were kept with a *CyO*, *twi-GFP* balancer. As control flies, we used the strain *w*^*1118*^ (Bloomington stock #3605). Wild-type and heterozygous, balanced *Pex19*^*ΔF7*^ flies were reared on standard fly food. *Pex2*^*HP35039*^, *Pex2*^*f01899*^, *Pex3*^*2*^, *Df(32)6262*, *Pex5*^*MI06050*^, *Pex10*^*MI04076*^, and *Df(2L) esc* ^*p3-0*^ were obtained from the Bloomington stock center; *Schlank*^*KINLS2*^ */FM7*, *Kr-GFP*, and *UAS-Schlank*^*aa1-138*^ flies were kindly provided by Reinhard Bauer. The latter insertion was recombined with the *Pex19*^*ΔF7*^ chromosome using standard *Drosophila* techniques. *Schlank*^*KINLS2*^*/Y; Pex19* ^*ΔF7/ΔF7*^ males were selected by absence of fluorescent markers. As control, we crossed *FM7*, *Kr-GFP/Y; pex19* ^*ΔF7*^*/CyO*, *twi-GFP* males with +; *Pex19*^*ΔF7*^*/CyO*, *twi-GFP* virgins and used the resulting homozygous *+/Y; Pex19* ^*ΔF7/ΔF7*^ males. For all assays, eggs were collected on apple juice agar plates (2% agar, 2.5% sucrose, 25% apple juice, 1.5% nipagin) with fresh yeast paste (42 g of yeast mixed with 10 ml of tap water), and first-instar larvae were transferred to fresh plates with yeast as control or yeast and supplements: 5% coconut oil, babassu oil, palm kernel oil, cocoa butter, rapeseed oil, olive oil, safflower oil, or broccoli seed oil. Oils of different manufacturers gave similar results. Except for babassu and palm kernel oil, which were refined and deodorized, native natural oils were used. Synthetic TAGs were from Sigma Aldrich. Nipagin was omitted from the apple juice agar plates for nipagin-free feeding assays. TAGs were purified from native coconut oil with a solid phase extraction column, and 5% TAGs were added to the yeast paste for feeding experiments. For the etomoxir assay, 100 μl of a 250-μM etomoxir (Sigma-Aldrich) solution was added to 500 mg of the yeast or coconut oil–containing yeast. For survival assays, 25 first-instar larvae were collected for each condition, and at least 5 independent experiments were conducted (exact numbers of *n* given in figure legends). The number of surviving pupae, adults (including pharates), and viable adults (survivors) that were able to move and lived at least 24 hours was counted.

### Larval locomotion assay

Five larvae at a time were put with a soft brush onto a prewarmed PBS-agar plate (10-mm petri dish) placed on top of millimeter graph paper and left for 1 minute to acclimate. Then, they were filmed using a Panasonic camcorder (HC-V380) for 40 seconds. Their movement was tracked manually by marking the position of the head every 2 seconds. The millimeter graph paper was used to scale the resulting image so that track lengths could be measured with ImageJ (Fiji). Larvae that did not move were excluded from analysis.

### Cell culture

Human fibroblast control and Δ19T cells were kept in Dulbecco’s modified eagle medium (DMEM, Gibco) with 10% FBS, 10,000 units of penicillin, and 10 mg streptomycin per ml. For coconut oil treatment, coconut oil was coupled to BSA at 37 °C, following an adapted protocol of Seahorse Bioscience for BSA-conjugated palmitate. In brief, 65.94 mg of coconut oil was added to 44 ml of 150 mM NaCl and stirred at 37 °C, and 11.2 ml was added to 14 ml BSA solution in 150 mM NaCl and stirred for 1 hour at 37 °C. For NEFA measurement, 1 × 10^5^ cells were seeded in 6-well plates and harvested after 48 hours. Cells were pelleted and washed with PBS. Cell pellets were treated like larval tissue (see Free fatty acid section). For experiments with coconut oil–BSA, cells were treated with 10% coconut oil–BSA 24 hours after seeding. Additionally, Δ19T cells were cultured with 10% coconut oil–BSA for several passages and seeded for NEFA measurement in normal medium. For live cell stainings, cells were seeded into 8-well slides for microscopy (Ibidi). After 48 hours, cells were washed carefully 3 times with PBS and stained with 50 nM TMRE for 15 minutes. Afterward, cells were washed again 3 times with PBS and immediately analyzed in the microscopy slide with PBS using a Zeiss LSM 710 with a 63× water objective (Plan-Apochromat, Zeiss).

### Imaging

We used an α-Schlank antibody [44], α-Lamin Dm0 (Developmental studies hybridoma bank, DSHB), and DAPI for (immune)stainings of *Drosophila* fat bodies. Secondary antibodies coupled to Alexa dyes were from molecular probes, and DAPI from Sigma-Aldrich. For (immune)histochemistry, we dissected tissue of interest from third-instar larvae. Tissue was fixed for 30 minutes in 3.7% formaldehyde and washed with PBT before and after incubation with primary antibody and Alexa dye–coupled secondary antibody. Tissue was mounted in Fluoromont G and analyzed using a Zeiss LSM 710 confocal microscope. Fat body cells were analyzed with a 25× water objective (Plan-Neofluar, Zeiss) and a pinhole of 1 Airy unit, and Malpighian tubules were analyzed with a 63× water objective (Plan-Apochromat, Zeiss) and a pinhole of 1 Airy unit. Human fibroblasts stained with TMRE were analyzed with a 63× water objective (Plan-Apochromat, Zeiss) and a pinhole of 1 Airy unit at a resolution of 1024 × 1024 pixels and at 1× zoom. For apoptosis assays, brains of adult flies were dissected and stained using an annexin V-FITC apoptosis detection kit (Sigma Aldrich) according to the manufacturer’s instructions. Imaging was done with identical imaging parameters for all conditions analyzed, using a 25× water lens (Plan-Neofluar, Zeiss) and a pinhole of 1 Airy unit on a Zeiss LSM710. Maximum intensity projections of 5 consecutive optical sections were generated for each genotype analyzed. For staining of mitochondria, 96-hour-old larvae or 5-day-old adults were dissected in ice-cold PBS, and their Malpighian tubules were stained for 20 minutes at RT with 50 nM TMRE (Sigma-Aldrich) in PBS according to the manufacturer’s protocol. The Malpighian tubules were then directly mounted in Fluoromount G and analyzed with a Zeiss LSM 710. Picture analysis and quantification were done using ImageJ. For quantification of Schlank subcellular localization, the corrected total cell fluorescence (CTCF) was measured with ImageJ using the Integrated Density and Area parameters. CTCF was calculated as CTCF = IntDen (Area × Mean gray value of background). The nuclear signal was determined by analyzing the area within the Lamin staining. For quantification of mitochondria, the particle size was measured with ImageJ. Each staining was done at least 5 times.

### Lipid profile

For quantification of FAMEs, 15 third-instar larvae were homogenized in 1 N MeHCl in a Precellys 24 homogenizer (peqlab). A minimum of *n* = 7 was analyzed for each condition. C15:0 and C27:0 standards were added, and samples were incubated for 45 minutes at 80 °C. Methyl esters were collected by addition of hexane and a 0.9% NaCl solution. The hexane phase was collected in a new glass vial and concentrated by vaporization. Samples were analyzed by GC/MS using an Agilent HP 6890 with an HP-5MS column. For FAME analysis in pupae, 100 pupae at *n* = 3 were processed as described above.

### Free fatty acids

NEFAs were measured by an adaptation of the copper-soap method [45]. In brief, 3 third-instar larvae were weighed and homogenized in 20 μl of 1 M phosphate buffer per mg tissue. Then, 25 μl of the supernatant were transferred to 500 μl of Chloroform/Heptane 4:3, and lipids were extracted by shaking the vial for 5 minutes. Unspecific background provoked by phospholipids was circumvented by addition of 23 mg of activated silicic acid. Next, 300 μl of the chloroform phase was transferred to 250 μl of Cu-TEA (copper-triethanolamine). After shaking and centrifuging, 150 μl of the organic phase was transferred to fresh cups. Liquid was evaporated in a 60 °C heat block, and lipids were dissolved in 100 μl of 100% ethanol. Copper was detected by complexation with a mixture of dicarbazone–dicarbazide, and the color intensity was measured in a 96-well plate at 550 nm in a TECAN plate reader. Each experiment was conducted at a minimum of 5 biological replicates.

### β-oxidation measurements

Six larvae per genotype were washed with PBS, and their weight was recorded for normalization purposes. The larvae were inverted in ice-cold PBS and permeabilized in ice-cold BIOPS buffer (2.77 mM CaK_2_EGTA, 7.23 mM K_2_EGTA, 5.77 mM Na_2_ATP, 6.56 mM MgCl_2_.6H_2_O, 20 mM taurine, 15 mM Na_2_.phosphocreatine, 20 mM imidazole, 0.5 mM DTT, 50 mM MES) containing 100 μg/mL saponin (fresh) at 4 °C with gentle rocking for 10 minutes. Then, the larvae were equilibriated in respiration medium (MiR05, 0.5 mM EGTA, 3 mM MgCl2*6H2O, 60 mM K-Lactobionate [lactobionic acid is dissolved in H2O, and pH is adjusted to pH 7.4 with KOH], 20 mM Taurine, 10 mM KH2PO4, 20 mM HEPES, 110 mM sucrose, 1 g/L fatty acid–free BSA) supplemented with 0.5 mM carnitine. The larvae were added into the oxygraph chambers, and oxygen concentration was brought to around 500 μM by using catalase and H_2_O_2_. After basal respiration was recorded, 5 μM palmitoyl-CoA was added to the chamber. Fatty acid β-oxidation was induced by adding complex I substrates, electron transfer flavoprotein (ETF) substrates, and ADP (10 mM proline, 10 mM pyruvate, 5 mM malate, 5 mM glutamate, 2 mM ADP, and 15 mM glycerol-3-phosphate). After that, etomoxir was added at the indicated concentrations to block fatty acid transfer into mitochondria via CPT I, thereby blocking β-oxidation and leaving complex I–dependent respiration. Finally, residual oxygen consumption (ROX) was measured by inhibiting complex III with antimycin A. All values were corrected for ROX. β-oxidation was calculated by subtracting etomoxir-resistant respiration from respiration in the presence of all substrates. Each measurement was repeated at least 3 times (biological replicates).

### Real-time qPCR

Whole RNA of third-instar larvae was isolated using TriFast reagent (peqlab). Tissue was homogenized using a Precellys 24 homogenizer (peqlab). Transcription to cDNA was performed using the Quantitect Reverse Transcription Kit (Quiagen). qPCR was performed with a CFX Connect cycler (biorad). A minus-RT was analyzed in a PCR for each cDNA. qPCR was performed with a CFX Connect cycler (biorad) using GoTaq SYBR Mix (Promega). Values were normalized against two housekeeping genes (actin5c and rp49) and against wild-type control (ΔΔC_q_). See Table 1 for primer sequences. Each experiment was repeated at least 5 times.

**Table 1:** qRT Primers.

**actin5c**	fwd rev	GTGCACCGCAAGTGCTTCTAA TGCTGCACTCCAAACTTCCAC
**rp49**	fwd rev	TCCTACCAGCTTCAAGATGAC CACGTTGTGCACCAGGAACT
**lip3**	fwd rev	ATCAAGTCCGCCCATCTTCT CTCTATGCCCAAATCCTGCT
**HexC**	fwd rev	GGCTTCACTTTTTCCTTCCC TTCCCACAATGACACCCAC
**yip2**	fwd rev	CGCAAAAGAACTGGGAGAAG TGCTCGTCCACCACAAAG
**Acsl**	fwd rev	GCCTCACACCCACCATTTT TCATTGTCATCATTCCGCAC
**IsoDH**	fwd rev	CAACTGATTCTGCCCTTCCT GGCGACTTCCACATCTTCTT
**mito Complex I**	fwd rev	ATCGATGACCAGCCAGTTGA GCTTGCTGGGATCGATCTTG

**Abbreviations**: Acsl, acyl-CoA synthetase long-chain; HexC, hexokinase C; IsoDH, isocitrate dehydrogenase; lip3, lipase 3; rp49, ribosomal protein 49; yip2, yippee-interacting protein 2

### Statistical analysis

We used the software GraphPad InStat for statistical analysis of our data. We applied the unpaired, two-tailed Student *t* test, assuming heteroscedasticity for single comparisons, and ANOVA with Tukey posttests and Bartlett’s test for homoscedasticity. Error bars represent standard deviation. Asterisks represent * *p* < 0.05, ** *p* < 0.01, *** *p* < 0.001.

## Supporting information

S1 Fig Fatty acid profile and neurodegeneration of *Pex19* mutants. (A) Representation of unsaturated and saturated nmol FAME/mg tissue. (B) Relative amount of FAMEs with the same chain length, calculated from the absolute concentration. Represented are, e.g., the sum of C14:0 and C14:1. (C) Total fatty acids from lipids (measured as FAMEs). (D) Negative geotaxis assay with 1-day-old adult flies. The time [seconds] in which the fly reached the 60 mm threshold was measured. Dots represent single experiments. Black bars represent median. Error bars represent SD. **p < 0.01; **p < 0.01. Corresponding raw data can be found in supplemental file S1 Data. FAME, fatty acid methyl ester.
(TIF)

S2 Fig Mitochondrial abundance, feeding behavior, and mitochondrial particle size. (A) Porin western blot from w- and *Pex19−/−* third-instar larvae fed on control or coconut oil diet. The picture shows the uncropped western blot with lysates from 96- and 120-hour-old larvae and detection with α-porin and α-tubulin as loading control. (B) Citrate synthase activity assay. (C) Larval feeding assay to determine yeast uptake. Bars represent quantification of the gut area stained with red yeast. (D) Quantification of mitochondrial particle size. TMRE-positive particle area was quantified with ImageJ. Error bars represent SD. *** *p* < 0.001. Corresponding raw data can be found in supplemental file S1 Data.
(TIF)

S3 Fig Basic characterization of the UAS Schlank^aa1–138^ construct, the ΔT19 human cell line, and prx19 RNAi *C*. *elegans*. (A) Third-instar larval fat body cells with clonal overexpression of Schlank^1–138^ and RFP. Scale bars as indicated. (B) Transcript levels of PEX19 in Δ19T fibroblasts compared to control fibroblasts. Fold change represents ΔΔCq. (C) Immunostaining of control and Δ19T cells with α-PEX3 (green) to show the absence of peroxisomes. Scale bars represent 10 μm. (D-E) Quantification of TMRE stainings of mitochondrial particle number and area in control fibroblasts. (F-G) Quantification of TMRE stainings of mitochondrial particle number and area in Δ19T fibroblasts. (H-K) Mito-GFP staining of *C*. *elegans* myo3:mtGFP muscle tissue, fed with prx19 RNAi knock-down bacteria. Scale bars represent 5 μm. Corresponding raw data can be found in supplemental file S1 Data. GFP, green fluorescent protein; prx19, putative peroxisomal biogenesis factor 19; RFP, red fluorescent protein; RNAi, RNA interference; TMRE, tetramethylrhodamine ethyl ester; UAS, upstream activating sequence.
(TIF)

S1 Text Supplemental methods and references. (DOCX)

S1 Data Raw data corresponding to Figs 1–6. (XLSX)

## Abbreviations

ABCD1: ATP-binding cassette family D transporter
ACC: acetyl-CoA carboxylase
Acsl: acyl-CoA synthetase long-chain
BSA: bovine serum albumin
CACT: carnitine acylcarnitine translocase
Colt: congested-like trachea
CPT I: carnitine palmitoyltransferase I
CRAT: carnitine O-acetyltransferase
CROT: carniting O-octanoyltransferase
CTCF: corrected total cell fluorescence
Cu-TEA: copper-triethanolamine
DMEM: Dulbecco’s modified eagle medium
ER: endoplasmic reticulum
ETF: electron transfer flavoprotein
FAME: fatty acid methyl ester
Fas: fatty acid synthase
FITC: fluorescein isothiocyanate
GAPDH: glyceraldehyde 3-phosphate dehydrogenase
GC/MS: gas chromatography/mass spectrometry
HexC: hexokinase C
Hnf4: hepatocyte nuclear factor 4
IsoDH: isocitrate dehydrogenase
KI: knock-in
LCFA: long-chain fatty acid
lip3: lipase 3
MCAD: medium-chain acyl-CoA dehydrogenase
MCFA: medium-chain fatty acid
NEFA: nonesterified fatty acid
ns: not significant
PBD: peroxisomal biogenesis disorder
PEX: peroxin
PGC1-α: peroxisome proliferator–activated receptor gamma coactivator 1-α
PMP: peroxisomal membrane protein
PPAR: peroxisome proliferator–activated receptor
RNAi: RNA interference
ROS: reactive oxygen species
ROX: residual oxygen consumption
rp49: ribosomal protein 49
SCAD: short-chain acyl-CoA dehydrogenase
SCFA: short-chain fatty acid
TAG: triacylglycerol
TCA: tricarboxylic acid
TEM: transmission electron microscopy
TMRE: tetramethylrhodamine ethyl ester
VLCAD: very-long-chain acyl-CoA dehydrogenase
VLCFA: very-long-chain fatty acid
X-ALD: X-linked adrenoleukodystrophy
yip2: yippee-interacting protein 2
