# Supplementary figures and images for: Dietary rescue of lipotoxicity-induced mitochondrial damage in Peroxin19 mutants
Source: PLoS Biol. 2018 Jun 19;16(6):e2004893. doi: 10.1371/journal.pbio.2004893 (PMC6025876; doi:10.1371/journal.pbio.2004893)

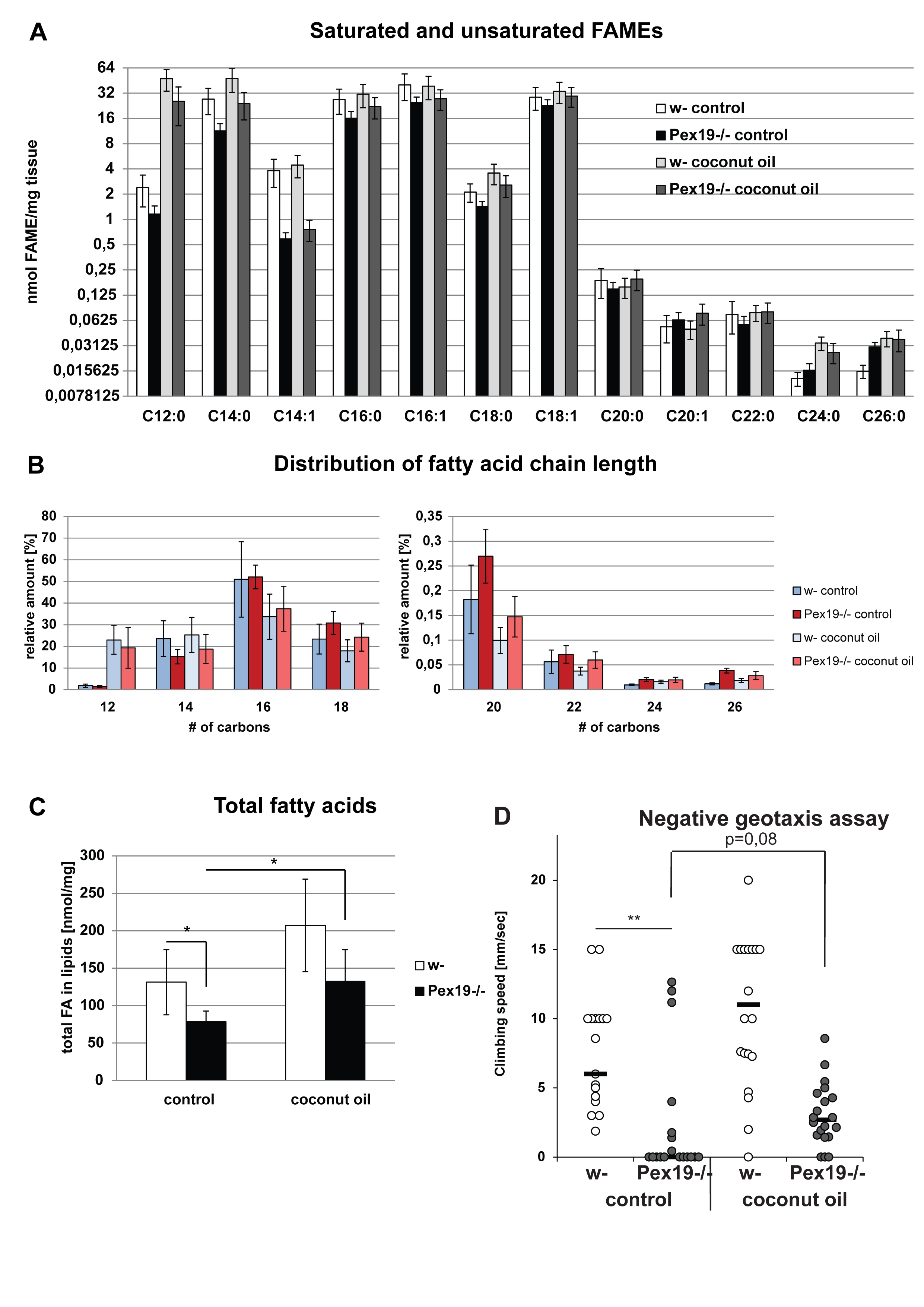

Supplement: S1 Fig — (A) Representation of unsaturated and saturated nmol FAME/mg tissue. (B) Relative amount of FAMEs with the same chain length, calculated from the absolute concentration. Represented are, e.g., the sum of C14:0 and C14:1. (C) Total fatty acids from lipids (measured as FAMEs). (D) Negative geotaxis assay with 1-day-old adult flies. The time [seconds] in which the fly reached the 60 mm threshold was measured. Dots represent single experiments. Black bars represent median. Error bars represent SD. **p < 0.01; **p < 0.01. Corresponding raw data can be found in supplemental file S1 Data. FAME, fatty acid methyl ester. (TIF) [file pbio.2004893.s001.tif]

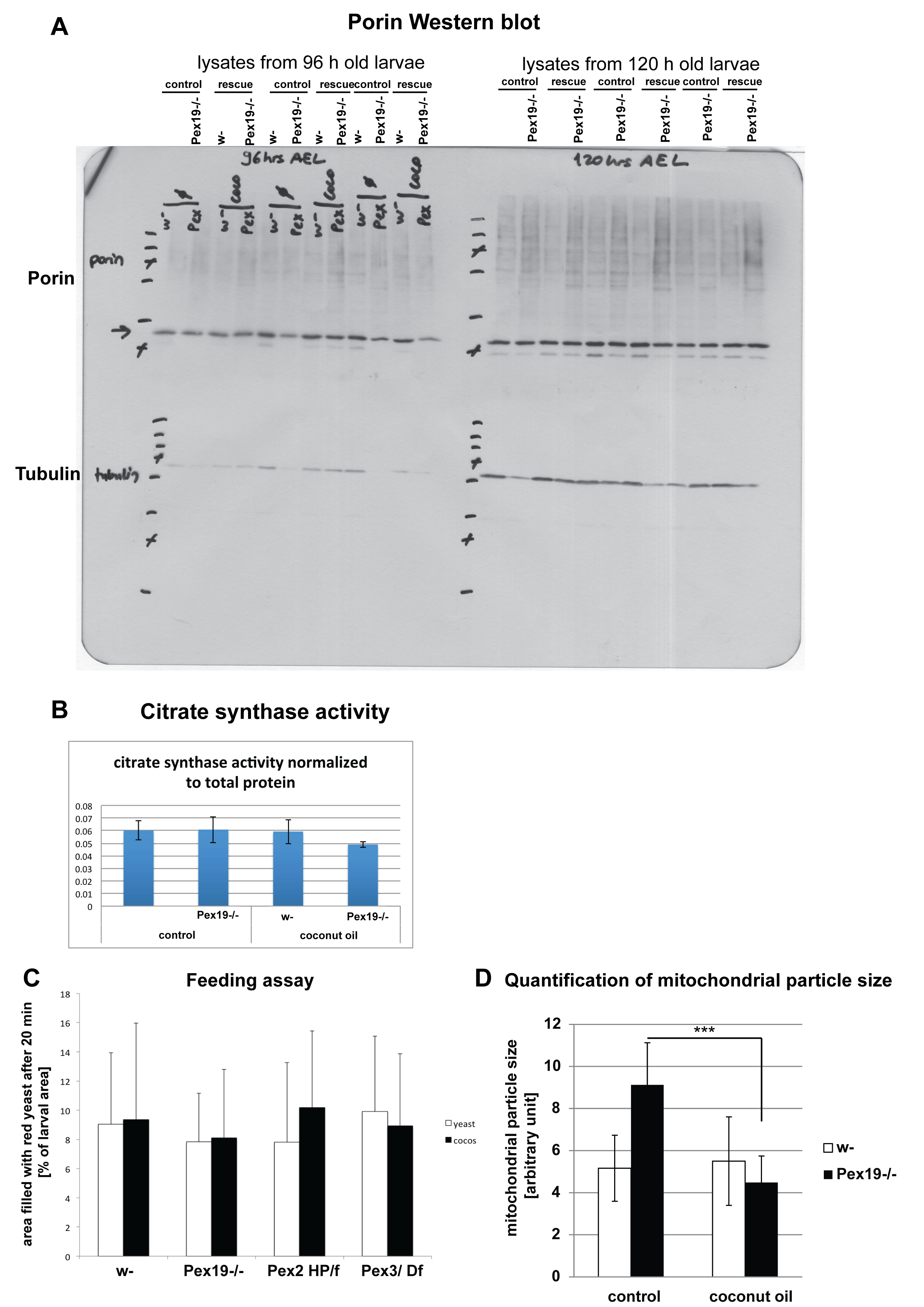

Supplement: S2 Fig — (A) Porin western blot from w- and Pex19−/− third-instar larvae fed on control or coconut oil diet. The picture shows the uncropped western blot with lysates from 96- and 120-hour-old larvae and detection with α-porin and α-tubulin as loading control. (B) Citrate synthase activity assay. (C) Larval feeding assay to determine yeast uptake. Bars represent quantification of the gut area stained with red yeast. (D) Quantification of mitochondrial particle size. TMRE-positive particle area was quantified with ImageJ. Error bars represent SD. *** p < 0.001. Corresponding raw data can be found in supplemental file S1 Data. (TIF) [file pbio.2004893.s002.tif]

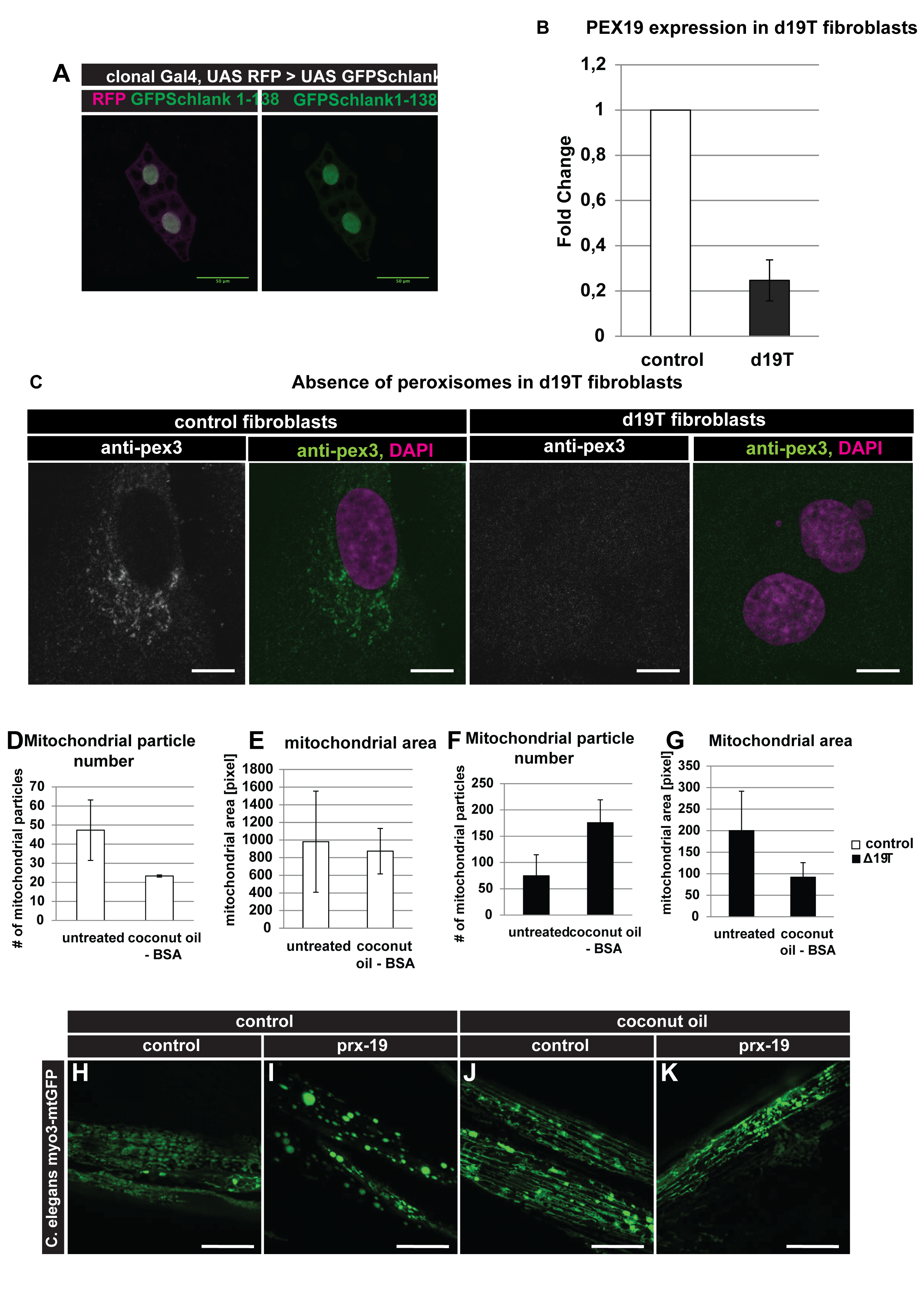

Supplement: S3 Fig — (A) Third-instar larval fat body cells with clonal overexpression of Schlank1–138 and RFP. Scale bars as indicated. (B) Transcript levels of PEX19 in Δ19T fibroblasts compared to control fibroblasts. Fold change represents ΔΔCq. (C) Immunostaining of control and Δ19T cells with α-PEX3 (green) to show the absence of peroxisomes. Scale bars represent 10 μm. (D-E) Quantification of TMRE stainings of mitochondrial particle number and area in control fibroblasts. (F-G) Quantification of TMRE stainings of mitochondrial particle number and area in Δ19T fibroblasts. (H-K) Mito-GFP staining of C. elegans myo3:mtGFP muscle tissue, fed with prx19 RNAi knock-down bacteria. Scale bars represent 5 μm. Corresponding raw data can be found in supplemental file S1 Data. GFP, green fluorescent protein; prx19, putative peroxisomal biogenesis factor 19; RFP, red fluorescent protein; RNAi, RNA interference; TMRE, tetramethylrhodamine ethyl ester; UAS, upstream activating sequence. (TIF) [file pbio.2004893.s003.tif]
