# Supplementary material for: Dietary rescue of lipotoxicity-induced mitochondrial damage in Peroxin19 mutants
Source: PLoS Biol. 2018 Jun 19;16(6):e2004893. doi: 10.1371/journal.pbio.2004893 (PMC6025876; doi:10.1371/journal.pbio.2004893)
Supplement: S1 Text — (DOCX) [file pbio.2004893.s004.docx]

**S1 Text. Supplemental methods and references**

**Supplemental Methods**

Yeast intake assay

Feeding behavior of larvae was analyzed as described in [1]. Briefly, washed larvae were starved in a Petri-dish with PBS-moistened tissue for 30min at RT. They were subsequently transferred onto yeast paste (as prepared for all feeding assays described in main manuscript Materials and Methods) containing 2% carmine on pre-warmed apple juice-agar plates and incubated for 20min at RT. Larvae were killed in 65°C hot water and photographed using a digital camera (Axiocam ICc 1, Zeiss) mounted on a binocular (Stemi 2000-CS, Zeiss). For each larva, amount of yeast ingested was calculated as area of the alimentary tract stained by red colored yeast as percentage of body surface area using the software ImageJ (Fiji).

Negative geotaxis assay

Single 1 day old adult flies were transferred into empty plastic vials. The same vials were re-used for all experiments. Time [sec] was measured when the fly reached the 60 mm line. Threshold was set at 60 sec. Each fly was tested 3 times.

C. elegans strains

Nematode strains used in this work were kindly provided by The Caenorhabditis Genetics Center (CGC, University of Minnesota).

Strains were maintained on NGM plates carrying a lawn of OP50 and kept at 20 °C. The following strains were used: N2 (ancestral) Bristol and SJ4103 zcIs14[myo-3p::GFP(mit)].

Imaging of C. elegans specimen was conducted by spinning disc microscopy which was performed using a fully motorized Nikon Eclipse Ti2000 inverted microscope with a Yokogawa CSU-x1 spinning disk, connected to an Andor iXON DU-897 EM-CCD camera. Images were acquired with a 100x oil-immersion objective (Nikon Plan Apo). Adult, gravid myo-3p::GFP(mit) worms were treated with sodium hypochlorite and eggs were plated on control (E. coli HT115 transformed with empty L4440 vector) and prx-19(RNAi) plates. Pure coconut oil was distributed on the bacterial lawn of treated animals. Worms were kept at 25 °C to prevent solidification of the coconut oil. At day of analysis, nematodes were paralyzed with 20 mM levamisole in M9 buffer and mounted on 2 % agarose pads on glass slides.

**Supplemental references**

[1] Schoofs A, Hückesfeld S, Pankratz MJ. Serotonergic network in the subesophageal zone modulates the motor pattern for food intake in Drosophila. J Insect Physiol. 2017 Jul 19. pii: S0022-1910(17)30115-4
